# Phonon Sideband Analysis and Near-Infrared Emission in Heavy Metal Oxide Glasses

**DOI:** 10.3390/ma14010121

**Published:** 2020-12-30

**Authors:** Joanna Pisarska, Wojciech A. Pisarski, Radosław Lisiecki, Witold Ryba-Romanowski

**Affiliations:** 1 Institute of Chemistry, University of Silesia, Szkolna 9 Street, 40-007 Katowice, Poland; wojciech.pisarski@us.edu.pl; 2 Institute of Low Temperature and Structure Research, Polish Academy of Sciences, Okólna 2 Street, 50-422 Wrocław, Poland; r.lisiecki@int.pan.wroc.pl (R.L.); w.ryba-romanowski@int.pan.wroc.pl (W.R.-R.)

**Keywords:** heavy metal glasses, phonon sideband, rare earth ions, luminescence

## Abstract

In this work, spectroscopic properties of europium and erbium ions in heavy metal oxide glasses have been studied. The phonon energy of the glass host was determined based on Eu^3+^ excitation spectra measurements. Near-IR emission spectra at 1550 nm related to ^4^I_13/2_ → ^4^I_15/2_ transition of erbium in heavy metal glasses were examined with special regards to luminescence bandwidth and measured lifetime. In particular, correlation between phonon energy and the measured lifetime ^4^I_13/2_ (Er^3+^) was proposed. The luminescence lifetime for the ^4^I_13/2_ upper laser state of erbium decreases with increasing phonon energy in glass matrices. Completely different results were obtained glass samples with europium ions, where the ^5^D_0_ lifetime increases with increasing phonon energy. Our investigations suggest that the values of measured ^5^D_0_ lifetime equal to radiative lifetimes for all heavy metal oxide glasses.

## 1. Introduction

Heavy Metal Oxide Glasses (HMOG) are classified as promising amorphous systems, for which chemical durability and thermal stability are high, light transmission is wide and refractive indices are quite large compared to similar glasses without PbO [1,2,3,4,5,6,7,8]. Heavy metal glass systems can be also quite easy synthesized because glass-forming compositional range is relatively wide. Among HMOG systems, PbO-GeO_2_ glasses are considered to be one of the most promising amorphous materials for numerous applications. Lead germanate glasses containing Ag nanoparticles are recommended in photonics and catalysis [9]. Low-loss lead germanate-based glasses are useful for mid-infrared fiber optics [10,11]. They are also interesting from the structural point of view. The presence of minima or maxima in physicochemical properties of germanate-based glasses under addition of various network-modifiers usually well correlate with the coordination change GeO_4_ tetrahedra ⟷ GeO_6_ octahedra, the formation of Ge-O-Ge bridging bonds and/or the creation of nonbridging oxygen atoms NBO’s [12,13]. The structural mechanism responsible for the germanate anomaly well-known in the literature was proposed for glasses based on PbO-GeO_2_ [14,15]. Similar to germanate glass systems, this anomalous composition dependence of physicochemical properties was also observed for borate-based glasses [16,17].

On the other hand, HMOG glasses are excellent matrices to accommodate rare earth ions. Very recently, emission properties of rare earth ions in glasses based on PbO-GeO_2_ [18,19], PbO-SiO_2_ [20], PbO-B_2_O_3_ [21,22] and PbO-P_2_O_5_ [23,24] were examined in detail and their results were published last year. The additional glass-former components such as Bi_2_O_3_ and TeO_2_ were also added in order to improve properties of heavy metal oxide glasses responsible for white light emission [25]. Special attention was devoted to rare earths in silicate glasses containing PbO, which emit near-infrared laser emission at about 2 µm [26,27,28,29,30].

In this work, we present spectroscopic properties of europium and erbium ions in heavy metal oxide glasses. Phonon sideband analysis based on the excitation spectra measurements of europium and near-infrared emission properties at 1550 nm corresponding to main ^4^I_13/2_ → ^4^I_15/2_ laser transition of erbium are reported. Emission spectra of rare earth ions in heavy metal oxide glasses have been also examined previously by us. In particular, spectroscopic properties of Eu^3+^ ions in lead borate glass [31], lead phosphate glass [32] and glasses based on PbO–Ga_2_O_3_–XO_2_, where X = Te, Ge, Si [33] have been systematically studied. Preliminary results for Er^3+^ ions in heavy metal oxide and oxyhalide glasses, including their near-infrared emission properties and up-conversion processes, have been presented in a conference paper [34]. Comparison of measured luminescence lifetimes ^4^I_13/2_ (Er^3+^) and ^5^D_0_ (Eu^3+^) gives rather unexpected results. While dependence of the ^4^I_13/2_ lifetime of erbium ions is well correlated with the phonon energies of the studied HMOG glass systems, the experimental results for luminescence decays from the ^5^D_0_ excited state of europium are completely different. These aspects have not been examined in detail before. They are discussed here.

## 2. Materials and Methods

HMOG glasses with general formula PbO-Ga_2_O_3_-Me_x_O_y_-Ln_2_O_3_ (Me denotes Ge, Si, P or B) were synthesized. In the studied glasses, rare earth oxides Ln_2_O_3_ were limited to Eu_2_O_3_ and Er_2_O_3_. The following samples: (a) 45PbO–9.5Ga_2_O_3_–45GeO_2_–0.5Ln_2_O_3_, (b) 45PbO–9.5Ga_2_O_3_–45SiO_2_–0.5Ln_2_O_3_, (c) 45PbO–9.5Ga_2_O_3_–45P_2_O_5_–0.5Ln_2_O_3_ as well as (d) 45PbO–9.5Ga_2_O_3_–45B_2_O_3_–0.5Ln_2_O_3_ (in mol%) were prepared. Metal oxides of high purity were mixed in an agate ball mill for 2 h and then melted (1100 °C/0.5 h).

Fully amorphous glass plates (dimension = 10 × 10 mm, thickness = 2 mm) confirmed by X-ray diffraction measurements were obtained. Differential scanning calorimeter DSC measurements (heating rate of 10 °C/min) were performed with SETARAM Labsys thermal analyzer (SETARAM Instrumentation, Caluire, France). Spectra measurements (excitation and emission) were carried out using a Continuum Model Surelite I optical parametric oscillator coupled with Nd:YAG laser (Continuum Surelite OPO and SLI-10 Nd:YAG laser, Santa Clara, CA, USA). Also, the laser system consists of 1-m double grating monochromator, photomultiplier, and Stanford SRS250 boxcar integrator. Resolution for spectra measurements was ±0.2 nm. Decay curves were measured using oscilloscope Tektronix TDS3052 (two channel color digital phosphor oscilloscope, 500 MHz, Tektronix Inc., Beaverton, OR, USA) with an accuracy of ±2 µs.

## 3. Results and Discussion

**Figure 1:**
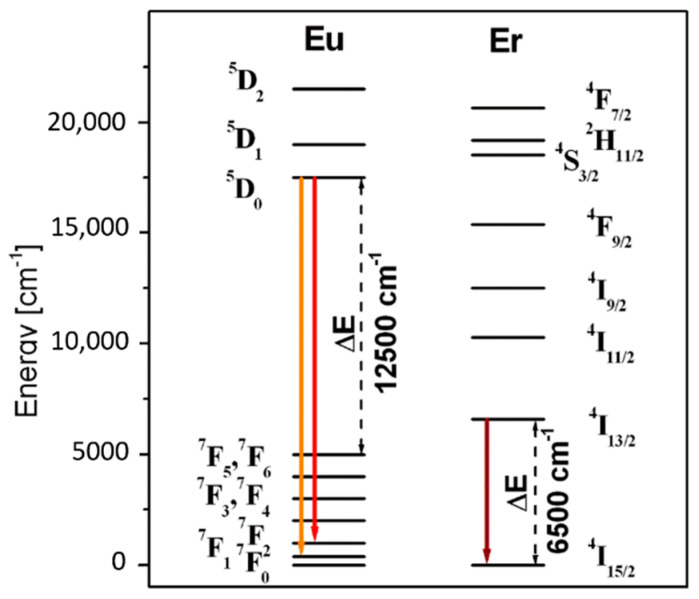
Energy level diagrams for trivalent europium and erbium ions.

HMOG glasses were prepared by traditional melt quenching technique. In all glass samples, the ratio of heavy metal oxide PbO to Me_x_O_y_ seems to be 1:1, whereas molar concentration of Ga_2_O_3_ and Ln_2_O_3_ is equal to 9.5% and 0.5%, respectively. Previous studies for boro-bismuth [35] and tellurite [36] glasses demonstrate significant role of Ga_2_O_3_ as a glass modifier, when its concentration is relatively low (usually less than 10 molar %). For the studied HMOG systems, the glass transition temperature T_g_ was determined based on DSC measurements. The values of T_g_ are changed in the following direction PbO-Ga_2_O_3_-B_2_O_3_ (440 °C) → PbO-Ga_2_O_3_-P_2_O_5_ (437 °C) → PbO-Ga_2_O_3_-SiO_2_ (429 °C) → PbO-Ga_2_O_3_-GeO_2_ (384 °C). Further investigations for lead borate glass [37] and lead phosphate glass [38] indicate that the glass transition temperatures are reduced with increasing PbF_2_ content. Ternary HMOG glasses based on PbO-Ga_2_O_3_-Me_x_O_y_ (Me = Ge, Si, P, B) were singly doped with Ln^3+^ (Ln = Eu, Er) in order to study their spectroscopic and emission properties. Figure 1 shows the energy level diagrams for rare earth (Eu^3+^ and Er^3+^) ions. All main emission lines corresponding to the ^5^D_0_ → ^7^F_1_ (orange line) and ^5^D_0_ → ^7^F_2_ (red line) transitions of europium ions and the ^4^I_13/2_ → ^4^I_15/2_ (near-IR line) transition of erbium ions are also indicated. It is worth noticing that the separation between the excited state ^5^D_0_ and the next lower-lying state ^7^F_6_ of europium ions (ΔE = 12,500 cm^−1^) is significantly larger than the ^4^I_13/2_–^4^I_15/2_ energy gap of erbium ions (ΔE = 6500 cm^−1^).

### 3.1. Phonon Sideband Analysis

**Figure 2:**
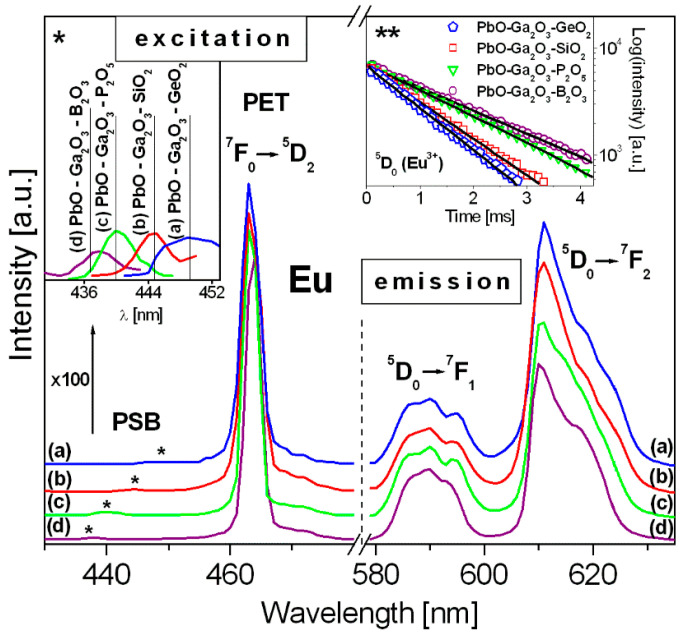
Excitation and luminescence spectra of HMOG glass systems containing europium ions. Phonon sidebands (PSB) and pure electronic transitions (PET) are shown. Insets present phonon sidebands (*) and decay curves for the ^5^D_0_ → ^7^F_2_ transition of Eu^3+^ (**).

Figure 2 presents excitation and emission spectra of the studied HMOG systems containing europium ions. Insets show phonon sidebands PSB (*) and decays from the ^5^D_0_ state of Eu^3+^ (**). Luminescence spectra of glass samples excited at 464 nm (^5^D_2_ state) consist of several narrowed bands characteristic for europium ions. Two main emission bands at about 590 nm and 615 nm correspond to ^5^D_0_ → ^7^F_1_ (orange) and ^5^D_0_ → ^7^F_2_ (red) electronic transitions [39,40,41,42]. The relative ratio of their integrated emission intensities known as R/O factor is changed in direction PbO-Ga_2_O_3_-B_2_O_3_ (2.32) → PbO-Ga_2_O_3_-P_2_O_5_ (2.41) → PbO-Ga_2_O_3_-SiO_2_ (3.00) → PbO-Ga_2_O_3_-GeO_2_ (3.06).

Luminescence decays from the ^5^D_0_ (Eu^3+^) state depend critically on the component Me_x_O_y_, i.e., GeO_2_, SiO_2_, P_2_O_5_ or B_2_O_3_, present in ternary HMOG glass. In fact, the ^5^D_0_ (Eu^3+^) measured lifetime diminishes in the following order: PbO-Ga_2_O_3_-B_2_O_3_ (2.05 ms) → PbO-Ga_2_O_3_-P_2_O_5_ (1.85 ms) → PbO-Ga_2_O_3_-SiO_2_ (1.27 ms) → PbO-Ga_2_O_3_-GeO_2_ (1.11 ms). To determine phonon energy of the host, the excitation spectra measurements of europium ions were successfully used. In 430–470 nm spectral range, two important bands of europium ions are located. They are assigned to pure electronic transition (PET) near 464 nm (^7^F_0_ → ^5^D_2_ transition) and well-known in the literature transition dependent on the host (observed usually in the ranges 440–460 nm), referred as phonon sideband PSB [43,44,45]. The phonon energy is difference between the positions of these bands (PSB-PET). Their values for the studied HMOG glasses are given in Table 1. At this moment, it should be mentioned that the obtained results are also consistent with the experimental values from the Raman spectra measurements. The values obtained by two independent methods are nearly the same and difference does not exceed ±3 cm^−1^. For example, the phonon energy for lead phosphate glass from Raman spectrum equal to 1120 cm^−1^ [46] is in a good agreement with the value obtained from the excitation spectrum measurement PSB-PET = 1117 cm^−1^ (Table 1). The same situation was observed for lead borate glass [31] and glasses based on PbO–Ga_2_O_3_–SiO_2_ and PbO–Ga_2_O_3_–GeO_2_ [33].

**Figure 3:**
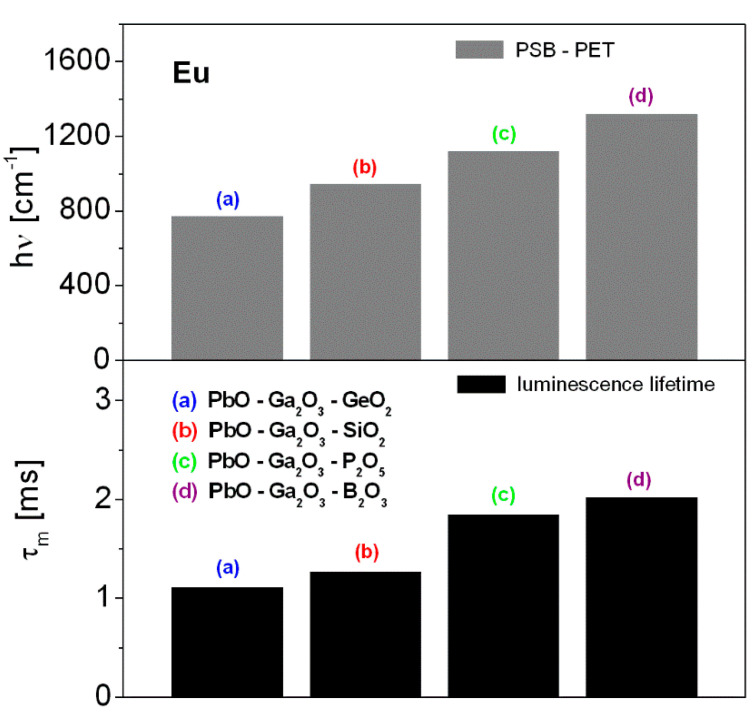
The influence of glass host composition on phonon energy and luminescence lifetime.

Like the ^5^D_0_ luminescence lifetime, the phonon energy of the HMOG glass systems diminishes in the order PbO-Ga_2_O_3_-B_2_O_3_ (1320 cm^−1^) → PbO-Ga_2_O_3_-P_2_O_5_ (1117 cm^−1^) → PbO-Ga_2_O_3_-SiO_2_ (950 cm^−1^) → PbO-Ga_2_O_3_-GeO_2_ (775 cm^−1^). The results are schematized in Figure 3.

### 3.2. Near-Infrared Emission

Our previous investigations revealed that low-phonon heavy metal oxide and oxyhalide glass systems containing erbium are excellent amorphous hosts for near-IR radiation and up-conversion luminescence applications. In contrast to glasses based on PbO-Ga_2_O_3_-GeO_2_ and PbO-Ga_2_O_3_-SiO_2_, the lack of up-conversion luminescence processes in PbO-Ga_2_O_3_-P_2_O_5_ and PbO-Ga_2_O_3_-B_2_O_3_ glass systems was confirmed, because P-O and B-O stretching vibrations are relatively large [34]. Further spectroscopic studies suggested that PbO-Ga_2_O_3_-GeO_2_ and PbO-Ga_2_O_3_-SiO_2_ glasses can be applied to up-conversion luminescence temperature sensors [47].

**Figure 4:**
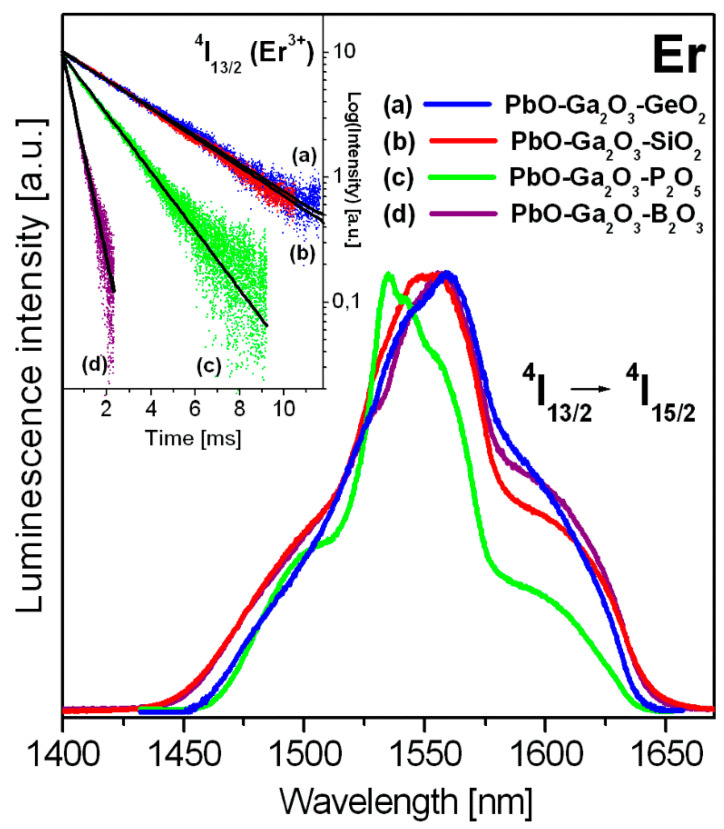
Normalized near-IR emission spectra for erbium ions in HMOG glass systems. Inset shows decays from the ^4^I_13/2_ state of erbium.

Figure 4 presents near-IR emission spectra measured for erbium ions in heavy metal oxide glass systems under excitation of ^4^F_7/2_ state by 488 nm laser line. Near-IR emission bands at about 1550 nm are assigned to main ^4^I_13/2_ → ^4^I_15/2_ transition of erbium. The inset shows emission decays from the ^4^I_13/2_ state of trivalent erbium.

Near-infrared emission spectra were normalized to compare the bandwidth for the ^4^I_13/2_ → ^4^I_15/2_ transition of erbium ions in HMOG glasses. The spectral bandwidth Δλ given as full width in half maximum (FWHM) was determined. Furthermore, the luminescence lifetimes of ^4^I_13/2_ state of erbium ions were calculated by fitting the luminescence decay curves. The bandwidth for the ^4^I_13/2_ → ^4^I_15/2_ transition of erbium ions is changed in the following direction PbO-Ga_2_O_3_-GeO_2_ → PbO-Ga_2_O_3_-SiO_2_ → PbO-Ga_2_O_3_-P_2_O_5_, except for PbO-Ga_2_O_3_-B_2_O_3_ glass system. The value of FWHM diminishes from lead germanate glass (nearly 88 nm) to lead phosphate glass (50 nm). Quite large spectral bandwidth (above 90 nm) for the ^4^I_13/2_ → ^4^I_15/2_ transition of erbium ions in PbO-Ga_2_O_3_-B_2_O_3_ glass system was observed. The relatively large bandwidth is strongly required for near-IR laser systems as well as glass fiber amplifiers. It confirms our previous experimental results obtained for oxide and oxyhalide lead borate-based glass systems containing erbium ions [34,48]. However, near-IR emission of erbium ions comes from multiple peaks and the spectral profiles are not simple Gaussian in shape. For the studied HMOG systems, differences in spectral bandwidth are relatively small when full width at 10% maximum (FW-10% max) will be considered.

**Figure 5:**
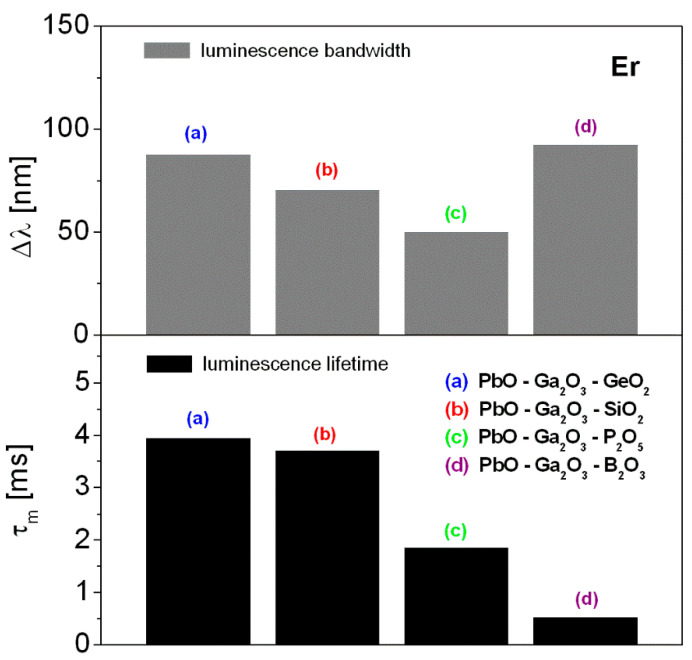
The influence of glass host composition on selected spectroscopic parameters (τ_m_, Δλ) determined for the ^4^I_13/2_ → ^4^I_15/2_ near-infrared laser transition of erbium.

Er^3+^-doped heavy metal oxide glass containing B_2_O_3_ exhibits interesting properties (broadband emission), but the emission decay from the ^4^I_13/2_ excited level of erbium is short when we compare to glass samples PbO-Ga_2_O_3_-Me_x_O_y_ (Me = Ge, Si or P). Both parameters, i.e., emission bandwidths and measured lifetimes are important spectroscopic factors and necessary to characterize glass materials with erbium for near-IR amplifiers [49]. The experimental results are schematized in Figure 5.

It is experimental evidence that the multiphonon relaxation rates of rare earth ions increase with increasing phonon energy in the following direction GeO_2_ → SiO_2_ → P_2_O_5_ → B_2_O_3_ [50,51]. Borate-based glass systems have the highest phonon energy, whereas germanate amorphous materials have the smallest phonon energy among the studied HMOG glass systems. Thus, measured lifetimes of rare earths usually reduce because multiphonon relaxation rates are higher. Our experimental results confirm this hypothesis. The measured ^4^I_13/2_ emission lifetimes of erbium ions are reduced from lead germanate glass (3.9 ms) to lead borate glass (0.5 ms) when phonon energy of the host increases.

**Figure 6:**
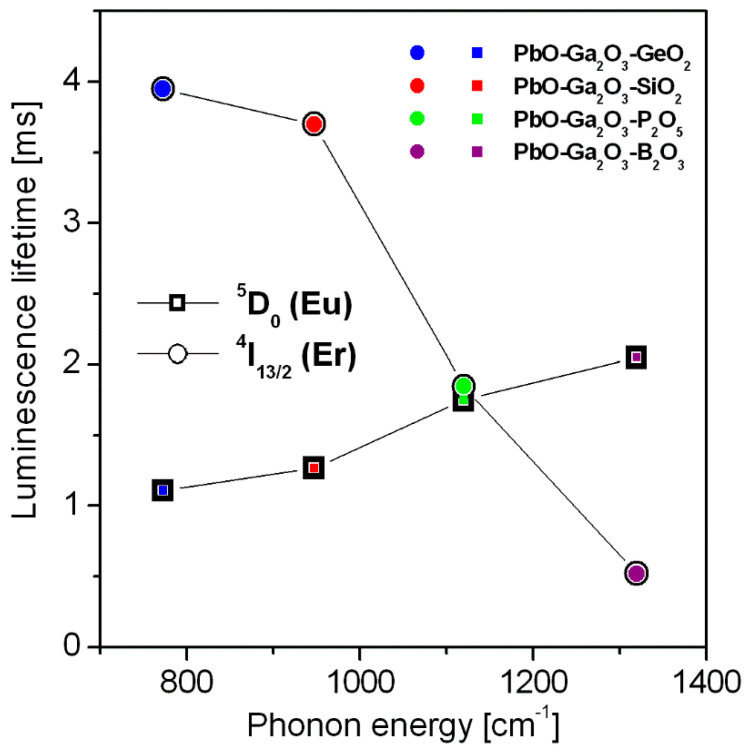
Lifetime for the ^5^D_0_ (Eu^3+^) and ^4^I_13/2_ (Er^3+^) excited states versus the phonon energy of the HMOG glass.

Correlation between chemical composition, glass structure and properties was shown by various experimental techniques [52,53,54]. In particular, structure-property correlations in heavy metal oxyfluoride glass systems have been explored in a series of lead fluorogermanate and lead fluoroborate glasses, where the increasing PbF_2_ content results in enhanced luminescence lifetimes of rare earths. These effects are much less apparent in lead fluoroborate than lead fluorogermanate glasses [55]. Interesting structure-property relationships are also observed for our HMOG glass samples. We can conclude from emission measurements that spectroscopic properties of trivalent rare earths are not only critically dependent on the glass host composition. The phonon sideband analysis demonstrates that phonon energy increases from PbO-Ga_2_O_3_-GeO_2_ to PbO-Ga_2_O_3_-B_2_O_3_ glass system (see Table 1). The activator concentration (Eu^3+^ and Er^3+^) for all studied glass samples is close to 0.5 mol%. The same experimental conditions were used for glass preparation, so the spectroscopic changes for rare earths (europium and erbium) are attributed to the change of the component Me_x_O_y_ (where Me = Ge, Si, P, B) in HMOG glasses. Here, our investigations were limited to glass samples, where the molar ratio of the main oxide components is the same, i.e., PbO:GeO_2_ = PbO:SiO_2_ = PbO:P_2_O_5_ = PbO:B_2_O_3_ = 1:1. In fact, the ratios of structural units (the same concentrations of Ge, Si, P and B atoms) are also important and cannot be ignored. Thus, nature of glasses with the same fractions of Me network-former units should be analyzed in the future. However, the main comparison is made between Eu^3+^ and Er^3+^ doped samples. Our spectroscopic analysis based on decay curves clearly indicates that the dependence of measured lifetimes for the excited states of rare earth ions on phonon energies of HMOG glass hosts is completely different for the ^4^I_13/2_ (Er^3+^) than the ^5^D_0_ (Eu^3+^). Figure 6 shows schematically measured emission lifetimes for the ^5^D_0_ (Eu^3+^) and ^4^I_13/2_ (Er^3+^) states as a function of the phonon energy of the HMOG system.

The measured lifetime for the ^4^I_13/2_ upper laser state of erbium decreases, whereas lifetime for the ^5^D_0_ state of europium increases with increasing phonon energy in glass matrices. Moreover, the change of the ^4^I_13/2_ measured lifetime by a factor of 8 when the phonon energy changes from 775 cm^−1^ to 1320 cm^−1^ is very high as compared to about two-fold increase of the ^5^D_0_ lifetime. This marked dissimilarity stems from the competition between radiative and multiphonon relaxation processes that remove the excitation of metastable levels of rare earth ions. The multiphonon relaxation consisting of simultaneous emission of the highest energy phonons in the lowest order process to cover the energy separation between a luminescent level and the next lower-lying energy level is consistent with the “energy gap law”. Considering the ^4^I_13/2_ metastable level located at about 6500 cm^−1^ above the next lower energy level ^4^I_15/2_ and phonon energies gathered in Table 1 we obtain the lowest order values for multiphonon relaxation of 8, 7, 6, and 5 for lead germanate, lead silicate, lead phosphate and lead borate glasses, respectively. We attribute observed dissimilarity of the ^4^I_13/2_ lifetime values to the contribution of multiphonon relaxation rates, especially in lead borate and lead phosphate glasses where the orders of the process are relatively low. The results are completely different for the same glass host matrices containing europium ions, where the energy gap between excited level ^5^D_0_ and the next lower level ^7^F_6_ is large (ΔE = 12500 cm^−1^) in comparison to separation between ^4^I_13/2_ and ^4^I_15/2_ levels of erbium ions (ΔE = 6500 cm^−1^) and non-radiative relaxation rates for rare earths are negligibly small. In fact, values of the order of multiphonon relaxation for the ^5^D_0_ state of Eu^3+^ amount to 16, 13, 11 and 10 for lead germanate, lead silicate, lead phosphate and lead borate glasses. Rates of multiphonon relaxation processes with orders of ten and higher are negligibly small. Therefore we can assume safely that measured luminescence lifetime values for europium-doped samples studied equal to radiative lifetimes of the ^5^D_0_ state of Eu^3+^.

## 4. Conclusions

Near-IR emission spectra of Er^3+^ ions in four HMOG glass hosts based on PbO–Ga_2_O_3_–GeO_2_, PbO–Ga_2_O_3_–SiO_2_, PbO–Ga_2_O_3_–P_2_O_5_ and PbO–Ga_2_O_3_–B_2_O_3_ are presented and discussed. The near-IR emission bands correspond to the ^4^I_13/2_ → ^4^I_15/2_ transition of erbium. In particular, spectroscopic parameters for erbium ions such as emission bandwidth and lifetime were determined. Correlation between luminescence lifetimes for erbium ions and phonon energies of the HMOG systems has been proposed. The measured lifetime for the ^4^I_13/2_ state of erbium ions decreases markedly with increasing phonon energy from PbO–Ga_2_O_3_–GeO_2_ to PbO–Ga_2_O_3_–B_2_O_3_ glass. Completely different results were obtained for the same glass host matrices containing europium ions, i.e., the measured lifetime for the ^5^D_0_ state of Eu^3+^ increases with increasing phonon energy. Owing to large ^5^D_0_–^7^F_6_ energy gap, non-radiative multiphonon relaxation rates are negligibly small and experimental values of luminescence lifetimes are equal to radiative lifetimes for all HMOG glasses containing europium ions.

## Figures and Tables

**Table 1:** Phonon energies (PSB-PET) determined from the excitation spectra measurements.

Heavy Metal Glass Host	PSB—PET [cm^−1^]
PbO-Ga_2_O_3_-GeO_2_	775
PbO-Ga_2_O_3_-SiO_2_	950
PbO-Ga_2_O_3_-P_2_O_5_	1117
PbO-Ga_2_O_3_-B_2_O_3_	1320

## Data Availability

The data presented in this study are available on request from the corresponding author.
